# A secondary exploratory study of associations between patient- and clinician-reported clinical outcomes and fidelity for four evidence-based psychosis treatments

**DOI:** 10.1186/s12888-025-07566-w

**Published:** 2025-11-28

**Authors:** Torleif Ruud, Jūratė Šaltytė Benth, Eva Biringer, Miriam Hartveit, Karin Drivenes, Kristin Heiervang, Tordis S. Høifødt, Vegard Ø. Haaland, Inge Joa, Jan Olav Johannessen, Karl Johan Johansen, Bjørn Stensrud, Espen Woldsengen Haugom, Hanne Clausen, Gary R. Bond

**Affiliations:** 1 Division of Mental Health Services, Akershus University Hospital, Lørenskog, Norway; 2 Institute of Clinical Medicine, University of Oslo, Oslo, Norway; 3 Health Services Research Unit, Akershus University Hospital, Lørenskog, Norway; 4 Section of Research and Innovation, Helse Fonna Health Trust, Haugesund, Norway; 5 Department of Health and Caring Sciences, Western Norway University of Applied Sciences, Stord, Norway; 6 Department of Global Public Health and Primary Care, University of Bergen, Bergen, Norway; 7 Division of Mental Health, Sørlandet Hospital, Kristiansand, Norway; 8 Centre for Medical Ethics, Institute for Health and Society, University of Oslo, Oslo, Norway; 9 University Hospital of Northern Norway, Tromsø, Norway; 10 Clinical Neuroscience Research Group, Department of Psychology, University of Oslo, Oslo, Norway; 11 TIPS Centre for Clinical Research in Psychosis, Psychiatric Division, Stavanger University Hospital, Stavanger, Norway; 12 Faculty of Health, Department of Public Health, University of Stavanger, Stavanger, Norway; 13 Mental Health Norway, Oslo, Norway; 14 Division of Mental Health, Innlandet Hospital Trust, Brumunddal, Norway; 15 Department of Psychology, Indiana University Indianapolis, Indianapolis, IN USA

**Keywords:** Evidence-based treatments, Fidelity, Psychosis, Patient-reported outcome, Clinician-reported outcome, Physical health care, Antipsychotic medication management, Family psychoeducation, Illness management and recovery

## Abstract

**Background:**

Implementation of evidence-based practices (EBPs), measured as fidelity to the EBP model, is generally expected to yield significant positive clinical outcomes. However, this association has only partially been established for EBPs used in psychosis treatment. From a cluster-randomized controlled trial (CRCT), we previously reported on the effects of implementation support for four EBPs for psychosis, using fidelity as the measure of implementation success. The current secondary, exploratory study used data from the non-blinded CRCT parent study to investigate the associations between patient- and clinician-reported outcomes and fidelity for these four EBPs.

**Methods:**

Clinical outcomes were measured in a cohort of 325 patients over three six-month periods. Primary outcomes were BASIS-24 (patient-reported) and HoNOS (clinician-reported). Secondary outcomes were selected subscales of these two measures. The EBPs were Physical Health Care, Antipsychotic Medication Management, Family Psychoeducation, and Illness Management and Recovery. In the CRCT, each of 39 clinical units across six health trusts selected two EBPs for implementation. Units were randomized to the intervention group (implementation support) for one EBP and the control group (written manual) for the other. Fidelity of the four EBPs was measured at baseline and every six months for 18 months. We analyzed the associations between outcomes and fidelity using linear mixed models.

**Results:**

BASIS-24 and HoNOS showed improvements for the total sample at 6 and 12 months, and two patient-reported subscales, Symptoms and Relationships, showed improvement at 6 months within two different EBP subsamples. However, no positive associations were found between secondary outcomes and EBP fidelity.

**Conclusions:**

Despite some improvements in primary and secondary outcomes over the first 6 to 12 months, we found no positive associations between outcomes and fidelity. Sample size, attrition, trial design, variance in variables, measurement properties, and low exposure, as well as interaction between such factors, might have contributed to our failure to find positive associations between outcomes and fidelity. Future studies of the association between outcomes and fidelity should involve large samples, use outcome and exposure measures closely related to the EBPs, and track cohorts from the beginning of treatment.

**Trial registrations:**

ClinicalTrials NCT03271242, retrospectively registered 31 August 2017.

**Supplementary Information:**

The online version contains supplementary material available at 10.1186/s12888-025-07566-w.

## Background

Evidence-based practices (EBPs) are defined as interventions that consistently lead to improved outcomes, as established by randomized controlled trials [1]. Within implementation science, fidelity measures the degree to which an implementation of an EBP adheres to the specific standards set by the EBP model [2]. Because fidelity is recognized as an intermediate measure of implementation success, it is widely used in implementation studies [3, 4]. Consequently, mental health services that maintain high fidelity to EBPs in psychosis treatment are expected to achieve positive patient outcomes. For several EBPs, specific fidelity scales have been developed to measure the essential components that account for the practice’s effectiveness [1].

Several biological and psychosocial EBPs have been documented to effectively reduce symptoms and problems associated with psychosis [5–7]. To investigate the real-world implementation of some EBPs, we conducted a cluster-randomized controlled trial (CRCT) assessing the impact of implementation support for four specific EBPs in psychosis treatment [8]: Physical Health Care, Antipsychotic Medication Management, Family Psychoeducation, and Illness Management and Recovery. Each participating clinical unit selected two of these EBPs and was randomized to serve as an intervention site for one EBP and a control site for the other. The primary outcome measure of the CRCT was the implementation success of these EBPs, quantified using a specific fidelity scale for each practice.

Prior evidence for the four EBPs is mixed. Physical Health Care is well-supported, with systematic reviews showing that physical exercise leads to significant improvements in negative psychosis symptoms, though effects on positive symptoms are often negligible [9–11]. Conversely, the literature on Antipsychotic Medication Management is scarce regarding multi-component, comprehensive approaches, as most studies treat its elements separately. However, limited evidence suggests that training in medication management can improve symptoms [12, 13]. Evidence for Family Psychoeducation is also limited, but improvement in clinician-reported symptoms have been reported [7]. Finally, Illness Management and Recovery has yielded mixed results, with some studies finding significant psychiatric symptom improvements [14–16] and others reporting non-significant findings [17–20].

Among 14 available fidelity scales for EBPs in psychosis treatment, eight have been reported to predict outcome [1]. However, the fidelity scales for the four specific EBPs utilized in our CRCT have not demonstrated this predictive capacity. Furthermore, to our knowledge, no study has yet examined the association between fidelity and outcome for any of these four EBPs. Therefore, we conducted this secondary exploratory study using data from the CRCT.

### Aims

The aim of this 18-month secondary exploratory study was to investigate whether patient- and clinician-reported outcomes in a cohort of patients with psychosis were associated with the fidelity of the implementing clinical units to four respective EBPs across intervention and control sites. To facilitate this analysis, we first examined outcomes for the total sample across the 6-, 12-, and 18-month follow-up periods, and explored selected relevant secondary outcomes for specific EBP subsamples across the three six-month follow-up assessments.

## Methods

### Design

This secondary, exploratory study followed a cohort of patients for 18 months to examine whether clinical changes were associated with EBP fidelity across three consecutive six-month periods. We utilized data from an 18-month CRCT, which originally assessed the effect of implementation support on the implementation of four EBPs in psychosis treatment [8]. The trial was retrospectively registered (ClinicalTrials NCT03271242, 31 August 2017). We adhered to the CONSORT Extension guidelines for CRCTs [21]. In the non-blinded CRCT, 39 clinical units each selected two of four core EBPs for implementation. A pairwise randomization design was used: each unit was assigned to be an intervention site (receiving implementation support) for one EBP and a control site (receiving only a written manual) for the other. Consequently, every unit served as an intervention site for one of the four EBPs.

### Context

Norwegian mental health services are predominantly public services provided by 19 health trusts that also manage general hospital services for all age groups [22, 23]. Adult mental health and substance abuse services consist of acute and other inpatient hospital units, and community mental health centers (CMHCs) with outpatient clinics, mobile teams, and local inpatient units [24]. Each CMHC serves a local catchment area and collaborates with general practitioners and municipal primary health and social care providers. All public inpatient and outpatient mental health services are free of charge, with the exception of an outpatient consultation fee. This fee is subject to an annual maximum, after which all subsequent consultations for the remainder of the year are free.

### Recruitment, samples and data collection

The leadership of the health trusts’ mental health services determined the participation of all CMHCs and other relevant clinical units. The study involved mental health clinics from six of the 19 Norwegian health trusts, which served 38% of the country’s population across both urban and rural settings. The 39 participating clinical units provided services to adults or adolescents with psychosis. These units included 26 CMHCs, 10 inpatient departments for adults with psychosis, and three combined outpatient and inpatient departments for adolescents.

We prospectively followed a total sample of 325 patients over the 18-month period, obtaining outcome data from both patients and clinicians at six-month intervals. This sample was recruited by clinicians across 32 of the 39 clinical units during a nine-month inclusion period. Seven clinical units did not recruit any patients. Local coordinators observed that in some units, the focus during the first months was mostly on learning and starting to implement the intervention EBP, while the parallel patient study with patient recruitment and data collection received less attention and efforts. Only patients who provided written informed consent were included. Subsamples for each EBP were comprised of patients receiving treatment at clinical units that had selected that specific EBP for implementation and had been randomized to either the intervention or control site for that EBP.

Table 1 presents patient characteristics and the distribution of intervention and control sites for EBP subsample at baseline. The sample size was progressively reduced at the 6, 12, and 18-month follow-up periods due to patient discharges, the death of 13 patients (confirmed by the Norwegian Cause of Death Registry), and other dropouts. Attrition rates for patient-completed questionnaires were 30%, 27%, and 25% compared to the last available data collection, while the reductions for clinician-reported ratings were 37%, 30%, and 22% at 6, 12, and 18 months, respectively. Descriptive statistics revealed only minor variations in characteristics across the remaining samples, though the rate of loss to follow-up was higher among males than females.

**Table 1:** Clinician-reported patient baseline characteristics and distribution of sites for each EBP

Samples	Baseline (*n* = 325)
Variables	*n* (%) / Mean (SD)
**Sex,** ***n*** **(%)**	
Male Female	191 (58.8) 134 (41.2)
**Age**,** mean (SD)**	39.9 (12.7)
**Age group**,** n (%)**	
16–19 20–29 30–39 40–49 50–59 60–69 70+	10 (3.1) 71 (21.8) 87 (26.8) 78 (24.0) 54 (16.6) 22 (6.8) 3 (0.9)
**Completed education**,** n (%)**	
Primary school uncompleted Primary school 7–10 years Secondary school Occupational education Higher education 1–3 years Higher education 4–6 years Other education Unknown (missing data)	9 (2.8) 96 (29.5) 82 (25.2) 54 (16.6) 40 (12.3) 23 (7.1) 10 (3.1) 11 (3.4)
**Total time of provided mental health services**,** n (%)**	
1 Less than 6 months 2 7–23 months 3 2–5 years 4 6–10 years 5 More than 10 years Unknown (missing data)	20 (6.2) 28 (8.6) 50 (15.4) 66 (20.3) 147 (45.2) 14 (4.3)
**Severity of mental illness (CGI)**,** mean (SD)**	4.03 (1.43)
Unknown (missing data), n (%)	16 (4.9)
**Severity of mental illness (CGI)**,** n (%)**	
1 No mental illness 2 Minimal mental illness 3 Mild mental illness 4 Moderate mental illness 5 Marked mental illness 6 Severe mental illness 7 Very severe mental illness Unknown (missing data)	4 (1.2) 58 (17.8) 47 (14.5) 77 (23.7) 67 (20.6) 52 (16.0) 4 (1.2) 16 (4.9)
**Functioning (PSF)**,** mean (SD)** Unknown (missing data), n (%)	4.02 (1.84) 8 (2.5)
**Diagnosis ICD-10**,** n (%)**	
Schizophrenia/paranoid psychosis Schizoaffective disorder Bipolar disorder Other Not reported	186 (57.2) 60 (18.5) 15 (4.6) 41 (12.6) 23 (7.1)
**Patients in intervention/control sites for each EBP**,** n / n**	
Physical Health Care Antipsychotic Medication Management Family Psychoeducation Illness Management and Recovery	108 / 122 64 / 57 39 / 74 114 / 72

CGI: Clinical Global Impression, PSF: Practical and Social Functioning, EBP: Evidence based practice

### Evidence-based practices, randomization and implementation support

**Evidence-based practices.** The four EBPs chosen for the trial were among those recommended in the Norwegian national clinical guidelines for psychosis treatment [25]. Two were biological practices (Physical Health Care, Antipsychotic Medication Management) that the clinical units were already providing without measuring quality. Two were psychosocial practices (Family Psychoeducation, Illness Management and Recovery) that were new to nearly all participating units.

The four EBPs were defined by the following core components: Physical Health Care involved monitoring cardiovascular risk factors, treating physical illnesses, and promoting physical fitness, a healthy diet, smoking cessation, and dental and oral health. Antipsychotic Medication Management encompassed shared decision-making, somatic assessment, medication choice and dosage, limiting polypharmacy, maintaining a current medication list, monitoring adherence and symptoms, monitoring side effects, and managing medication discontinuation. Family Psychoeducation provided families with education, skills training, and support through meetings and workshops over the course of a year or longer. Illness Management and Recovery focused on psychoeducation (to enhance illness understanding), prevention (to reduce relapses and hospitalizations), behavioral training (to improve medication adherence), coping skills training (to mitigate symptoms), and social training (to strengthen social support). Previous papers have elaborated on these four EBPs in greater detail [26–29].

**Randomization.** In the CRCT, all clinical units received detailed descriptions of the four EBPs and selected two for implementation. They agreed to be randomized to serve as intervention site (receiving implementation support) for one practice and control site (receiving only written guidelines) for the other. Of the units, 26 selected Physical Health Care, 17 selected Antipsychotic Medication Management, 14 selected Family Psychoeducation, and 21 selected Illness Management and Recovery. Stratified randomization, performed by an external statistician, ensured an even distribution between the intervention and control conditions for each of the six possible pairs of practices.

**The implementing support** provided to intervention sites included a toolkit for the EBP. Clinicians participated in EBP-specific training, and implementation facilitators conducted frequent site visits. Additional telephone supervision was provided for the two psychosocial practices. Furthermore, clinicians received feedback every six months based on the fidelity assessment and the results from a questionnaire to the staff about their experiences with the implementation process [30]. The intervention period spanned 18 months, lasting from September 2, 2016, to February 28, 2018. More details about the implementation support are available in the paper discussing its impact [8].

### Measures

#### Outcome measures

For *primary patient-reported outcome measure*, we used the mean total score from the Behavior and Symptom Identification Scale 24 (BASIS-24). BASIS-24 has demonstrated acceptable reliability and validity for patients experiencing psychosis, as well as sensitivity to change [31–34]. As *secondary patient-reported outcome measures* for the EBP subsamples and for analyzing associations with fidelity, we used two BASIS-24 subscales: the Psychosis subscale (5 items), which relates to Antipsychotic Medication Management, and the Relationships subscale (6 items), which relates to Family Psychoeducation and Illness Management and Recovery. These subscales were more relevant to the respective EBPs than the total BASIS-24, which consists of several dimensions. The BASIS-24 contains 24 items rated on a scale of 0 to 4, with higher scores indicating more serious symptoms or issues. The Norwegian translation was approved by the license holder following adjustments based on back-translation. We calculated mean scores for the total scale and the selected subscales according to the instructions and item weights specified in the BASIS-24 manual [35]. In our study, the BASIS-24 total scale showed fair internal consistency (Cronbach’s alpha) across all time points: 0.71 at baseline, 0.72 at 6 months, 0.71 at 12 months, and 0.69 at 18 months. The alpha range was 0.75 to 0.76 for the Psychosis subscale and 0.72 to 0.80 for the Relationships subscale.

For the *primary clinician-reported outcome measure*, we used the mean total score of the Health of the Nation Outcome Scale (HoNOS) [36]. HoNOS is a widely recognized rating scale to address core psychiatric problems while remaining brief enough for clinicians to complete amid their busy schedules. As *secondary clinician-reported outcome measures* for the EBP subsamples and for analyzing associations with fidelity, we used two HoNOS subscales: the Symptoms subscale (3 items), related to Antipsychotic Medication Management, and the Functioning subscale (2 items), related to Family Psychoeducation and Illness Management and Recovery. These subscales were more relevant to the respective EBPs than the total HoNOS scale which consists of several dimensions. HoNOS was familiar to the clinicians, as it constituted part of the standard data set used by health trusts for routine reporting to the Norwegian Patient Register. Clinicians were required to undertake 45-minutes e-learning training on proper HoNOS rating. Developed especially for patients with serious mental illness, HoNOS consists of 12 items rated on a scale from 0 = “No problem” to 4 = “Severe or very severe problem” [36].

Because the selected secondary outcome measures were more relevant to the content of the EBPs and their fidelity scales, we chose to use these secondary outcomes—rather than the primary outcomes (total scales)—for the exploratory analyses of associations between patient- and clinician-reported outcomes and fidelity.

Missing values for the BASIS-24 and HoNOS scores at 6 months (*n* = 19 for BASIS-24 and *n* = 13 for HoNOS) and at 12 months (*n* = 15 for BASIS-24 and *n* = 11 for HoNOS) were interpolated from existing values at adjacent time points. The interpolation method used the previous time point’s value plus half of the difference between the previous and subsequent time points. Overall, the interpolated data constituted 8% of the BASIS-24 data and 7% of the HoNOS data.

#### Explanatory variables

**Fidelity of the four EBPs** was assessed by trained fidelity assessors using a specific fidelity scale for each EBP. Two of these scales (Family Psychoeducation Fidelity Scale, Illness Management and Recovery Fidelity Scale) had been developed and utilized in prior studies [26, 27]. The other two scales (Physical Health Care Fidelity Scale, Antipsychotic Medication Management Fidelity Scale) were developed for our CRCT due to the absence of established fidelity scales for these EBPs [28, 29]. All four fidelity scales exhibited adequate psychometric properties (interrater reliability, internal consistency, sensitivity to change, feasibility) as corroborated in the articles cited above.

Fidelity assessment at 6 months was conducted in March-April 2017, at 12 months in September-October 2017, and at 18 months in March-April 2018. Two trained assessors rated fidelity for the two practices being implemented in each clinical unit. The assessors conducted in-person site visits, rating fidelity independently and resolving any discrepancies through consensus. The fidelity visits included interviews with managers and clinicians, reviews of written material for each EBPs, and review of documentations found in 10 randomly selected patient records for Physical Health Care and Antipsychotic Medication Management.

The fidelity achieved during the CRCT is reported in detail in a previous paper [8]. Summarized, the increase in fidelity scores (within range 1–5) over 18 months was significantly greater for intervention sites compared to control sites for three of the four EBPs. Effect sizes (Cohen’s d) for increase in difference between intervention and control sites of mean fidelity scores were 2.24 for Illness Management and Recovery, 0.68 for Physical Health Care, 0.71 for Antipsychotic Medication Management, and 0.27 for Family Psychoeducation. Most improvements occurred during the first 12 months.

**Clinician-recorded patients’ exposure to each EBP.** The two biological interventions are administered to most patients with psychosis, while the two psychosocial interventions are provided by a limited number of clinicians to a restricted selection of patients. We therefore identified the need for a measure of how much each patient was exposed to the respective intervention to adjust for this in the analysis of associations between outcome and fidelity. Penetration (exposure) has been defined as one of eight implementation outcomes [3].

Exposure for the 0–6 months period was registered by clinicians as time-period and number of sessions attended. Because clinicians found it too time-consuming to extract these data from patient records, this registration was modified at 12 and 18 months. Instead, clinicians then rated exposure to components of each EBP on a five-point ordinal scale considering the scope, regularity and systematicity of the EBP: 0=‘Nothing in this period’, 1=’a little/occasionally, not systematic’, 2=‘Partially systematic/short time (weeks)’, 3=’Quite systematic/longer time (months)’, and 4=‘Systematic/regularly, all the time’. To ensure uniformity, data reported at 6 months were transformed to align with the ordinal scale used at 12 and 18 months. This measure with the ordinal scale was developed by the research group for this study but it was not tested for validity or interrater reliability. An English translation of the registration form for exposure is found in the Supplementary Material, and the calculation of the estimated exposure for each EBP (mean of the relevant items) is shown below the registration form in the Supplementary Material. Table A in Supplementary Material shows descriptive statistics of the estimated exposure to each of the four EBPs during each six-months period as reported by the clinicians. The exposure was highest for the two biological EBPs and lowest for the two psychosocial EBPs.

**Patient characteristics**. From data collected at patient inclusion in the trial, we selected five baseline patient characteristics to adjust for in our analyses of associations between secondary outcomes and fidelity: age, sex, severity of mental illness, functioning, and duration of contact with mental health services. Severity was rated by clinicians using the Clinical Global Impression (CGI) [37]. CGI is a single seven-point scale ranging from 1 = “normal, not ill” to 7 = “among the most severely ill”, and it is shown to be reliable and valid [37]. Functioning was rated by clinicians interviewing patients using the revised Practical and Social Functioning (PSF) scale version 2 with 32 items and a five-point scale ranging from 1 = “completely incorrect” to 5 = “completely correct”. PSF has been shown to have adequate internal consistency [38], and Cronbach’s alpha was 0.69 at baseline in our study. The total duration of mental health services provided to the patient was rated by clinicians on an ordinal variable with a six-point scale defined for the trial as a proxy measure of mental illness duration (see Table 1 for the ordinal scale).

### Data collection

Patients reported on their mental health by completing the BASIS-24 questionnaire. Clinicians assessed the patients’ mental problems using the HoNOS and collected additional baseline information on patient characteristics, as well as rated the exposure to the EBPs. Clinical unit personnel collected the data at the time of patient inclusion (baseline, or 0 months) and again at 6, 12, and 18 months thereafter. The inclusion period spanned nine months, running from June 1, 2016, to February 28, 2017.

### Data analysis

We reported descriptive statistics of clinician-reported baseline patient characteristics for the total sample, as well as the distribution of each EBP subsample in intervention and control sites. Primary and secondary outcomes were presented by means and standard deviations (SDs) at each time point and each period within intervention and control sites, respectively.

To analyze the changes in BASIS-24 and HoNOS (primary outcomes) for the total sample from baseline to 6, 12, and 18 months, we estimated linear mixed models with fixed effects for time periods (coded as dummies 0–6, 0–12, and 0–18). Random effects for patients nested within unit and health trust were included if necessary, according to Akaike’s Information Criterion (AIC). As a higher score in the outcome variables indicated more severe problems, a positive difference from the start to the end of the period indicated improvement, while a negative difference indicated deterioration. We reported regression coefficients (RCs) with 95% confidence intervals (CIs) and effect sizes (Cohen’s d), adjusting the models for baseline patient characteristics including age, sex, duration of treatment, severity of mental illness, and level of functioning. To test if those lost to follow-up at 6, 12 and 18 months were different in primary outcomes than those remaining in the study (“drop-out analyses”), we used independent samples t-test on the primary outcomes for these groups six months earlier.

To analyze changes in the subscales of BASIS-24 and HoNOS (secondary outcomes) across the six-month periods for the EBP subsamples, we applied an independent samples t-test at each period and reported the results as mean differences between the two groups with corresponding 95% CIs and p-values. As with the primary outcomes, a positive difference from the start to the end of a period indicated an improvement and a negative difference a deterioration.

To analyze associations between the subscales of BASIS-24 and HoNOS (secondary outcomes) and the clinical unit’s fidelity to the EBP model, we estimated linear mixed models with random intercepts for patients nested within unit and health trust whenever needed according to AIC. We included fixed effects for periods (coded as dummies 0–6, 6–12, and 12–18), unit group (intervention vs. control) and fidelity at the end of each period, and all two- and three-way interactions. We applied AIC to reduce the models for excessive interactions. Random effects for health trust and clinical unit level were considered, but not included as it did not enhance model fit according to AIC. All adjusted models included baseline patient characteristics (age, sex, duration of treatment, severity of mental illness, level of functioning), and patient’s exposure to the practice during the previous six months. Standard residual diagnostics were performed. The results were presented as regression coefficients and standard errors.

The inference results were presented along with p-values for the sake of transparency. Given the exploratory nature of the study, the reported p-values should be viewed as providing preliminary insights rather than definitive evidence of significance. As such, they warrant cautious interpretation and calls for further confirmation in future studies. We used SPSS for Windows version 28 for descriptive analyses and STATA version 17 for linear mixed model analyses.

## Results

### Patient- and clinician-reported outcomes

Primary patient- and clinician-reported outcomes are presented descriptively in Table 2. According to the results of the linear mixed model, also presented in Table 2, there was an improvement with small effect size in BASIS-24 and HoNOS scores for the total patient sample at 6 and 12 months but not at 18 months. The additional t-test revealed that for HoNOS, the 117 patients remaining in the study at 12 months showed more severe problems (*p* < 0.001) at 6 months than the 58 patients lost to follow-up at 12 months. However, no such differences were observed 6 months earlier between patients remaining or lost to follow-up at either 6 or 18 months. For BASIS-24, no differences were found at any time point between remaining patients and those lost to follow-up.

**Table 2:** Primary outcomes (BASIS-24 and HoNOS) for the total sample at 6, 12 and 18 months. Descriptive statistics by time point and results from adjusted linear mixed models

	**Descriptive statistics in primary outcomes at each time point**					
	**BASIS-24 mean total score**			**HoNOS mean total score**		
**Time point**	**N**	**Mean (SD)**		**N**	**Mean (SD)**	
Month 0 Month 6 Month 12 Month 18	309 224 153 116	1.23 (0.61) 1.12 (0.65) 1.06 (0.66) 1.09 (0.66)		274 175 125 87	0.78 (0.47) 0.70 (0.47) 0.66 (0.45) 0.66 (0.44)	
	***Results of adjusted linear mixed model for trend in primary outcome variables***					
	**BASIS-24 mean total score** ^**1**^			**HoNOS mean total score** ^**2**^		
**Time point**	**RC (95% CI)**	**p-value**	**Effect size**: **Cohen’s d**	**RC (95% CI)**	**p-value**	**Effect size**: **Cohen’s d** ^**3**^
Intercept	0.91 (0.56; 1.25)	< 0.001		0.39 (0.13; 0.65)	0.004	
Month 0 – ref.	0	-		0		
Month 6	-0.10 (-0.18; -0.02)	0.010	0.16	-0.07 (-0.13; -0.01)	0.022	0.21
Month 12	-0.14 (-0.23; -0.05)	0.003	0.10	-0.07 (-0.13; -0.0004)	0.049	0.22
Month 18	-0.08 (-0.18; 0.02)	0.129	0.13	-0.07 (-0.14; 0.01)	0.086	0.22

The numbers are adjusted for patient characteristics at baseline (age, sex, years in treatment in mental health services, severity of mental illness, and functioning)

BASIS-24: Behavior and Symptom Identification Scale. HoNOS: Health of the Nation Outcome Scales

RC: Regression coefficient, CI: Confidence interval

^1^Model with random effects for patients nested within HF

^2^Model with random effects for patients nested within unit

As shown for secondary outcomes for EBP subsamples in Table 3, patients at intervention sites for the Antipsychotic Medication Management reported greater improvement in the BASIS-24 Symptoms subscale compared to those at control sites during the initial six months. Likewise, patients receiving the Illness Management and Recovery intervention showed better outcomes on the BASIS-24 Relationships subscale over the first six-month period but with a deterioration between six and twelve months. Clinician-rated HoNOS Symptoms and Functioning subscales did not show any improvement for the Antipsychotic Medication Management or the Illness Management and Recovery groups, respectively.

**Table 3:** Secondary outcomes for EBP subsamples. Descriptive statistics and results of **independent samples t-test of the differences between intervention and control sites**

Secondary patient-reported outcomes: BASIS-24 subscales															
**Outcome**		**Psychosis subscale** **for Antipsychotic Medication Management**							**Relationship subscale for** **Illness Management and Recovery**						
		**Intervention sites**				**Control sites**			**Intervention sites**				**Control sites**		
**Period**		**n**		**Mean (SD)**		**N**		**Mean (SD)**	**n**		**Mean (SD)**		**n**		**Mean (SD)**
0–6 6–12 12–18		47 32 21		0.19 (0.83) 0.08 (0.70) -0.41 (0.90)		37 28 22		-0.28 (0.84) 0.37 (0.99) 0.01 (0.95)	75 50 36		0.47 (1.00) -0.19 (1.21) 0.25 (1.07)		53 38 28		0.001 (0.93) 0.34 (0.88) -0.11 (1.43)
		**Mean diff. (95% CI)**						**p-value**	**Mean diff. (95% CI)**						**p-value**
0–6 6–12 12–18		0.47 (0.10; 0.83) -0.29 (-0.73; 0.15) -0.41 (-0.98; 0.16)						0.013 0.190 0.152	0.47 (0.12; 0.81) -0.52 (-0.99; -0.06) 0.36 (-0.26; 0.99)						0.009 0.027 0.248
***Secondary clinician-reported outcomes: HoNOS subscales***															
**Outcome**	**Symptoms subscale for** **Antipsychotic Medication Management**								**Functioning subscale for** **Illness Management and Recovery**						
	**Intervention sites Control sites**								**Intervention sites Control sites**						
**Period**	**n**		**Mean (SD)**		**N**		**Mean (SD)**		**n**	**Mean (SD)**		**n**		**Mean (SD)**	
0–6 6–12 12–18	36 30 13		0.08 (0.64) 0.15 (0.91) 0.10 (0.72)		34 23 16		0.005 (0.50) -0.05 (0.72) 0.10 (0.72)		53 40 26	0.21 (0.65) 0.07 (1.11) -0.01 (0.80)		26 18 16		0.17 (0.81) 0.06 (0.77) -0.23 (1.17)	
	**Mean diff. (95% CI)**						**p-value**		**Mean diff. (95% CI)**					**p-value**	
0–6 6–12 12–18	0.08 (-0.20; 0.35) 0.20 (-0.26; 0.66) -0.16 (-0.73; 0.41)						0.569 0.389 0.581		0.03 (-0.30; 0.37) 0.01 (-0.57; 0.59) 0.22 (-0.39; 0.84)					0.839 0.964 0.463	

BASIS-24: Behavior and Symptom Identification Scale. HoNOS: Health of the Nation Outcome Scales

CI: Confidence interval

### Association between secondary outcomes and fidelity

Table 4 shows the associations between the BASIS-24 and HoNOS subscales (secondary outcomes) and fidelity to the EBP models. Overall, fidelity was not associated with the BASIS-24 or HoNOS subscales. The only exception was the Symptoms subscale in relation to Antipsychotic Medication Management, where higher fidelity was associated with less improvement in the intervention group.

**Table 4:** Associations between subscales of secondary outcomes and fidelity, stratified by unit groups

Patient-reported outcomes for BASIS-24 subscales and evidence-based practices. Adjusted linear mixed models *						
	**Psychosis and Antipsychotic Medication Management** **(*n* = 183**,** 10–11 sites)**		**Relationships and Family Psychoeducation** **(*n* = 161**,** 9–11 sites)**		**Relationships and Illness Management and Recovery (*n* = 235**,** 12–16 sites)**	
**Variables / Periods**	**RC (SE)**	**p**	**RC (SE)**	**p**	**RC (SE)**	**p**
**Intercept**	-2.26 (1.21)	0.061	0.08 (1.05)	0.941	0.34 (0.61)	0.576
**Period** (0–6 – ref.)						
6–12 12–18	0.71 (0.22) 0.21 (0.24)	0.001 0.384	-0.29 (0.23) -0.29 (0.26)	0.215 0.276	0.16 (0.25) -0.03 (0.28)	0.527 0.921
**Unit group** (Control – ref.)						
Intervention sites	0.31 (0.21)	0.128	-0.35 (0.29)	0.226	0.22 (0.29)	0.450
**Period x Group**						
6–12	-0.98 (0.31)	0.002	0.42 (0.39)	0.281	-0.72 (0.32)	0.026
12–18	-0.95 (0.33)	0.004	0.19 (0.44)	0.655	-0.16 (0.36)	0.662
**Fidelity**	0.49 (0.30)	0.099	-0.01 (0.09)	0.880	0.07 (0.08)	0.381
Clinician-reported outcomes for HoNOS subscales and evidence-based practices. Adjusted linear mixed models*						
	**Symptoms and Antipsychotic Medication Management (*n* = 146**,** 6–8 sites)**		**Functioning and Family Psychoeducation** **(*n* = 114**,** 8–10 sites)**		**Functioning and Illness Management and Recovery** **(*n* = 154**,** 10–13 sites)**	
**Variables / Periods**	**RC (SE)**	**p**	**RC (SE)**	**p**	**RC (SE)**	**p**
**Intercept**	-1.58 (1.41)	0.264	-1.95 (1.11)	0.079	0.32 (0.70)	0.655
**Period** (0–6 – ref.)						
6–12 12–18	0.02 (0.22) -0.05 (0.24)	0.937 0.834	-0.26 (0.26) -0.52 (0.30)	0.310 0.085	-0.32 (0.28) -0.06 (0.30)	0.250 0.829
**Unit group** (Control – ref.)						
Intervention sites	6.81 (2.56)	0.008	-0.17 (0.30)	0.558	0.10 (0.25)	0.687
**Period x Group**						
6–12	0.59 (0.34)	0.083	0.29 (0.39)	0.460	0.13 (0.34)	0.699
12–18	0.90 (0.51)	0.078	0.60 (0.46)	0.197	-0.27 (0.38)	0.473
**Fidelity**	0.40 (0.37)	0.279	0.03 (0.09)	0.717	-0.02 (0.07)	0.824
**Group x Fidelity**						
Intervention sites	-2.19 (0.84)	0.009				

RC: Regression coefficient, SE: Standard error. The numbers are adjusted for patient characteristics at baseline (age, sex, years in treatment in mental health services, severity of mental illness, and functioning) and exposures to EBPs that are not shown in the table

## Discussion

### The results seen in relation to other studies

The improvements in primary outcomes for the total sample observed in the first 12 months and the improvement of secondary outcomes for two EBP subsamples in the first 6 months align with mixed results from previous studies on similar treatments in mental health services. For example, two pragmatic trials on training providers in medication management showed improved symptoms for patients with schizophrenia after 6 to 9 months [12, 13]. Of three RCTs on family psychoeducation, only one reported symptom improvement at the end of treatment, with another showed improvement at a 12-month follow-up [39–41]. Studies on Illness Management and Recovery also yielded inconsistent findings: two pre-post studies and one RCT found evidence of improvement [14–16], whereas three separate RCTs, all with moderate to large sample sizes, reported non-significant results [18–20].

Studies investigating associations between clinical outcomes in psychosis and fidelity to EBP models are limited. We are not aware of any such studies specifically addressing any of the four EBPs in our CRCT. However, the lack of associations between clinical outcomes and fidelity in our study partially aligns with the limited results in studies on such associations in other EBPs for severe mental disorders. For instance, one study found no significant differences in psychiatric symptoms between high- and low-fidelity programs [42]. Another study on dual disorder programs showed limited associations between fidelity and psychosis symptoms [43]. Finally, a recent study on case management with modest sample size and low variance in fidelity failed to identify a significant association between fidelity and psychiatric symptoms [44]. We have no explanation to the negative association found between the Symptom subscale and Antipsychotic Medication Management, and this may be a random finding due to factors discussed below. The association between outcome and fidelity is one of the important areas of research on EBP fidelity, and studies should measure both outcomes and fidelity [1].

### Factors potentially contributing to the lack of positive results

Several factors might have contributed to our failure to find significant positive associations between clinical outcomes and fidelity, as well as the limited results in patient- and clinician-reported outcomes.

**Sample sizes and attrition.** The subsamples used to analyze secondary outcomes were limited to patients from units designated as intervention or control sites for specific EBPs. Both these subsamples and the total sample diminished over time, potentially impacting the results [45]. In line with CONSORT recommendations, we examined variations in changes over time and found mostly small variations. This suggests that the limited improvement might be due to the low statistical power inherent in small samples [46]. While linear mixed models partially helped control for attrition, influence on the outcomes likely persisted [47].

Patients may have been lost to follow-up for various reasons, such as discharge or non-completion of questionnaires. We did not collect data on the reasons, which hindered a thorough assessment of these. Furthermore, attrition effects might have varied based on differences between patients who remain in treatment and those lost to follow-up [47]. It is probable that patients who remained in mental health services for a longer duration had more severe and enduring mental health issues. This is supported by the observed differences in illness severity at 6 months between patients who remained in the study and those who were lost to follow-up at 12 months. A paper on social factors and personal recovery, showed that the patients remaining in our study at 18 months were more often women and older than those lost to follow-up, and most likely represented patients with more severe or chronic problems that had not been referred to primary care due to improvement [48].

**Exposure to the EBPs.** A central explanation for the lack of associations may be the limited exposure of patients to the EBP. Inclusion in the data analyses did not require that patients had received the specific intervention implemented at their site. As a result, a considerable number of patients in the EBP subsamples had little or no exposure to the EBPs, as shown in the descriptive data in Table A in the Supplementary Material. Even though our analyses adjusted for exposure, the measurement of exposure may have been hampered by uneven reporting and underreporting by clinicians with varying knowledge of treatments provided during the last six months. This misalignment means that fidelity assessed at the service level may primarily reflect organizational implementation capacity rather than the actual treatment delivered to the included patients. The absence of a more systematic linkage between individual exposure and clinical outcomes likely diluted any fidelity–outcome associations and represents a methodological limitation of the study.

In addition to the limited and uneven exposure, the small EBP subsamples and the attrition over time further reduced the statistical power to detect associations. The combination of modest exposure, varying sample sizes, and attrition likely contributed to random variation overshadowing potential intervention effects. These issues are not unique to the present study but highlight the challenges of evaluating complex, multicomponent interventions under real-world service conditions. Among 400 implementation studies reported in a scoping review, only 15% had used a measure of penetration (exposure) [4].

Our exposure measure was a general ordinal scale trying to integrate scope, regularity and systematicity of the delivery of the EBP to make it easier for clinicians to provide information. However, exposure measures more specific to elements of each EBP are probably needed. As an example, patients’ attendance to Illness Management and Recovery session are recorded, and a recent review found that attendance represented a recurring determinant for outcome across the studies [49]. As shown in the Supplementary Material Table A and known from clinical practice, there are some differences in provision of the four EBPs. The two biological EBPs are provided by a large group of mental health workers to many patients with psychosis, while the two psychosocial EBPs typically are provided by a few specially trained clinicians to a smaller, selected group of patients. Scheduled specific sessions provided by clinicians in specific roles or with specific competencies are easier to register and measure than ad hoc informal conversations between patients and several staff members providing motivation and support. Furthermore, our ordinal exposure scale, which has unknown psychometric properties, may not have provided a valid and reliable measurement of the actual patient exposure. A possible solution for future studies may be specific systematic information recorded regularly in electronic patient records that may be extracted as clinical feedback and for research without burden for clinicians.

**Inclusion process and inclusion period.** The inclusion process may have influenced clinical outcomes because we included both patients in ongoing treatment and newly referred patients. This approach differs from studies tracking cohorts from treatment initiation to completion. Many were recruited during early treatment phases, where treatment effects on psychiatric issues are often more pronounced, although we lack supporting data to confirm this effect [50]. A study on proportion of patients with psychosis who was willing to take part in research found that the patients who were approached had better clinical outcomes than those who were not, suggesting that clinicians were more likely to approach patients who were less ill [51]. This might be the same in our CRCT.

All patients were followed up every six months for 18 months, provided they were not discharged and remained willing to participate. However, the nine months inclusion period introduced variability in the timing of patient outcome data relative to fidelity data. This meant that outcome data for patients recruited late in the period were analyzed against a fidelity assessment that had been completed several months earlier. With variations in the clinical course of different patients, it is difficult to know how this might have influenced the possibility to detect positive associations between outcomes and fidelity.

**Informants and outcome measures.** Differences between informants and characteristics of the outcome measures may have influenced the results. Combining patient- and clinician-reported outcomes is standard because their roles, perspectives, and access to information may describe different and supplementary aspects of the patient’s condition. Consequently, discrepancies in reported results may be expected. A recent study found that the correlation pattern between patient- and clinician-reported measures showed a three-layer structure, representing a continuum from inner experience (e.g., subjective quality of life, only known to the patient) to the external presentation of experiences (e.g., symptom severity, known to both patient and clinician) [52]. The lack of changes in secondary clinician-reported outcomes might also be partially due to a lower sensitivity to change of the HoNOS subscales, which have fewer items, providing lower resolution than the BASIS-24 subscales [53, 54]. We could not find any other study using both BASIS-24 and HoNOS as outcome measures. In our study, the correlation between the two total scales was moderately strong, while correlations between their subscales ranged from weak to strong [55].

**The low variance in fidelity** likely influenced the results by limiting the possibility of detecting a positive association with outcomes. This limitation persisted even during the initial 6–12 months, when the changes in both outcome and fidelity were greatest. Furthermore, even for Illness Management and Recovery, which demonstrated a large effect size for the increase in fidelity at intervention sites compared to control sites, we found no association between outcome and fidelity. This finding is likely attributable to the low exposure of patients to the EBP, as discussed above. We cannot rule out that outcome might have been associated with subgroups of fidelity scale items with larger variance than the total scale and more closely related to the selected secondary outcomes.

**The complex design of the CRCT**, where each site served as both an intervention site for one EBP and a control site for another, was chosen to facilitate the inclusion of four EBPs in the CRCT with EBP fidelity as the primary outcome. As noted previously, comparing four distinct EBPs within the same study revealed differences among them that impact implementation and fidelity. However, for this current secondary exploratory study, this complex design may have reduced the ability to detect positive associations between outcomes and fidelity. Specifically, the fact that all sites were intervention sites for one EBP might have led to greater overall engagement among both clinicians and patients than if some units had been purely passive control sites. Although three of the EBPs demonstrated a significantly higher increase in fidelity at intervention sites compared to control sites, these differences did not translate into an association with outcome. Additionally, we did not account for other treatments provided to patients, which may have had a more substantial impact on clinical outcomes than the designated EBPs.

### Strengths and limitations

The study’s strengths include the use of data from a CRCT, and the application of well-established outcome measures with adequate psychometric properties. The EBPs selected were core practices recommended in national guidelines for treating individuals with psychosis. Furthermore, the fidelity of these practices was assessed by independent raters using specific fidelity scales, which also demonstrated adequate psychometric properties. Finally, data analysis utilized linear mixed models, as recommended for longitudinal studies to limit the impact of participant attrition.

However, several limitations were present. A substantial proportion of patients was lost to follow-up between measurement points, and this attrition may have influenced the results. Patient recruitment and data collection from both patients and clinicians were carried out by clinicians and health personnel as part of their busy clinical duties, and with unknown inter-rater reliability. Furthermore, the EBP subsamples were small. The measure used for exposure to EBPs had unknown measurement properties. Clinicians’ assessments regarding patients’ exposure to each EBP required modifications after the data collection at six months. Patients were included during various illness phases, and variations existed in the timing of outcome data collection relative to fidelity assessments. The design of the parent CRCT was less suitable for the needs of the current secondary exploratory study. Finally, data was not collected on the reasons for patients being lost to follow-up.

### Conclusions and implications

There was improvement in both primary outcomes for the total sample over the first 12 months, along with improvement in two patient-reported secondary outcomes for EBP subsamples over the first 6 months. Crucially, we found no association between outcomes and fidelity for any of the EBP subsamples. Our failure to find associations between outcomes and fidelity might be attributed to several factors, including sample size, attrition, trial design, variance in variables and measurement properties, as well as interactions between these factors.

These findings emphasize the importance of aligning fidelity assessments with actual patient-level exposure when evaluating implementation outcomes. Future studies investigating the association between outcomes and EBP fidelity should involve large samples, utilize measurement scales with strong psychometric properties, track patient cohorts from the beginning of treatment, and ensure a close correspondence between the delivery of EBPs and the measurement of both fidelity and clinical change.

## Supplementary Information

Below is the link to the electronic supplementary material.

Supplementary Material 1

## Data Availability

The dataset used and analyzed during the current study is available from the corresponding author on reasonable request.

## Abbreviations

AIC: Akaike’s Information Criterion
BASIS-24: Behavior and Symptom Identification Scale
CAMHS: Child and adolescent mental health services
CGI: Clinical Global Impression scale
CI: Confidence interval
CMHC: Community mental health center
CONSORT: Consolidated Standards of Reporting Trials
CRCT: Cluster-randomized controlled trial
EBP: Evidence-based practice
HoNOS: Health of the Nation Outcome Scales
PSF: Practical and Social Functioning Scale
RC: Regression coefficient
RCT: Randomized controlled trial
