# Supplementary material for: A secondary exploratory study of associations between patient- and clinician-reported clinical outcomes and fidelity for four evidence-based psychosis treatments
Source: BMC Psychiatry. 2025 Nov 28;25:1134. doi: 10.1186/s12888-025-07566-w (PMC12661844; doi:10.1186/s12888-025-07566-w)
Supplement: Supplementary file 1 — Supplementary Material 1 [file 12888_2025_7566_MOESM1_ESM.pdf]

Supplementary material: Page completed by clinicians on patients' exposure to treatment

All treatment the patient has received in the mental health services during the last 6 months

These data will be linked to the data on implementation of practices in the units, and is therefore crucial for assessing whether patient patients' clinical course and satisfaction are associated with implementation.

Instruction: Register all treatment the patient has received in the division/clinic in the last 6 months. Mark one box for each treatment (on each line). Consider the scope, regularity and systematicity of the intervention.

**What the patient has participated in the last 6 months (one mark on each line)**	**0 Nothing during this period**	**1 A little / occasionally, not systematic**	**2 Partially systematic / short time (weeks)**	**3 Fairly systematic / longer time (months)**	**4 Systematic / regularly, all the time**
1. Organized physical ctivity/exercise/ training	☐	☐	☐	☐	☐
2. Courses/training in diet/cooking/ nutrition	☐	☐	☐	☐	☐
3. Examination/follow-up of physical illness/risk	☐	☐	☐	☐	☐
4. Programs of smoking cessation/reduction of smoking	☐	☐	☐	☐	☐
5. Follow-up of dental health	☐	☐	☐	☐	☐
6. Antipsychotic medication and follow-up	☐	☐	☐	☐	☐
7. Meetings with family (patient and family)	☐	☐	☐	☐	☐
8. Multi-family group (patients and family)	☐	☐	☐	☐	☐
9. Network meetings	☐	☐	☐	☐	☐
10. Life skills training (Illness Management and Recovery)	☐	☐	☐	☐	☐
11. Life skills training (other program:)	☐	☐	☐	☐	☐
12. Training in self-care and independence	☐	☐	☐	☐	☐
13. Individual Placement and Support (supported employment)	☐	☐	☐	☐	☐
14. Cognitive behavioral therapy	☐	☐	☐	☐	☐
15. Other forms of psychotherapy	☐	☐	☐	☐	☐
16. Work on substance use problems	☐	☐	☐	☐	☐
17. Group therapy/activity	☐	☐	☐	☐	☐
18.Other treatment:	☐	☐	☐	☐	☐

Additional information in this supplementary material: Calculation of the measure of exposure for each of the four evidence-based practices based on the rating of most of the items above:

- Exposure to Physical health care: mean of items 1 – 5.
- Exposure to Antipsychotic Medication Management: Item 6.
- Exposure to Family Psychoeducation: Mean of items 7-9.
- Exposure to Illness Management and Recovery: mean of items 10-12.

Supplementary Material

Table A. Clinicians' ratings of patients' exposure* to each evidence-based practice during the three six months periods. Descriptive statistics for each EBP subsample.

Frequence table of distribution of exposure* to each evidence-based practice

Time period and exposure: N (% of EBP subsample at baseline)	Physical Health Care n (%)	Antipsychotic Medication Management n (%)	Family Psycho-education n (%)	Illness Management and Recovery n (%)
	Evidence-based practice			
**0 – 6 months**				
0 Nothing in this period	34 (14.8)	16 (13.2)	57 (50.9)	98 (53.3)
1 A little / occasionally, not systematic	71 (31.0)	41 (33.9)	17 (15.2)	18 (9.8)
2 Partially systematic, short time (weeks)	34 (14.8)	13 (10.7)	0 (0.0)	4 (2.2)
3 Quite systematic/longer time (months)	23 (10.0)	3 (2.5)	1 (0.9)	1 (0.5)
4 Systematic/regularly, all the time	5 (2.2)	13 (10.7)	6 (5.4)	11 (6.0)
Missing information	62 (27.1)	35 (28.9)	31 (27.7)	52 (28.3)
**6 – 12 months**				
0 Nothing in this period	35 (15.3)	9 (7.4)	39 (34.8)	61 (33.2)
1 A little / occasionally, not systematic	26 (11.4)	5 (4.1)	9 (8.0)	7 (3.8)
2 Partially systematic, short time (weeks)	28 (12.2)	6 (5.0)	3 (2.7)	4 (2.2)
3 Quite systematic/longer time (months)	22 (9.6)	14 (11.6)	3 (2.7)	11 (6.0)
4 Systematic/regularly, all the time	19 (8.2)	32 (26.4)	9 (8.0)	10 (5.4)
Missing information	99 (43.2)	55 (45.5)	49 (43.8)	91 (49.5)
**12 – 18 months**				
0 Nothing in this period	25 (10.9)	9 (7.4)	27 (24.1)	45 (24.5)
1 A little / occasionally, not systematic	20 (8.7)	4 (3.3)	13 (11.6)	8 (4.3)
2 Partially systematic, short time (weeks)	17 (7.4)	1 (0.8)	3 (2.7)	6 (3.3)
3 Quite systematic/longer time (months)	22 (9.6)	4 (3.3)	3 (2.7)	2 (1.1)
4 Systematic/regularly, all the time	11 (4.8)	28 (23.1)	4 (3.6)	10 (5.4)
Missing information	134 (58.5)	75 (62.0)	62 (55.4)	113 (61.4)

Descriptive statistics of exposure* to each evidence-based practice

Time period	Physical Health Care		Antipsychotic Medication Management		Family Psychoeducation		Illness Management and Recovery	
	N	Mean (SD)	n	Mean (SD)	n	Mean (SD)	n	Mean (SD)
0 – 6 months	167	1.37 (1.37)	86	1.49 (1.27)	81	0.54 (1.11)	132	0.55 (1.17)
6 – 12 months	130	1.72 (1.72)	66	2.83 (1.45)	63	0.95 (1.48)	93	0.95 (1.47)
12 – 18 months	95	1.73 (1.73)	46	2.83 (1.66)	50	0.88 (1.26)	71	0.93 (1.46)

*) Response scale for the ordinal variable on exposure:
0 = "Nothing in this period", 1 = "A little, occasionally, not systematic", 2 = "Partial systematic, short time (weeks)", 3 = "Quite systematic/longer time (months)", 4 = "Systematic/regularly, all the time".
